# Molecular physiology unlocks the mystery that relates cognitive impairment with the retina in schizophrenia and autism spectrum disorders: a perspective review

**DOI:** 10.3389/fpsyt.2024.1495017

**Published:** 2024-11-11

**Authors:** Sarah Al-Mazidi

**Affiliations:** ^1^ Department of Anatomy and Physiology, Imam Muhammad ibn Saud Islamic University, Riyadh, Saudi Arabia; ^2^ College of Medicine, Imam Mohammad Ibn Saud Islamic University, Riyadh, Saudi Arabia

**Keywords:** autism, schizophrenia, cognition, retina, OCT, biomarkers

## Abstract

Schizophrenia and Autism spectrum disorders (SSD and ASD) are neurodevelopmental disorders involving cognitive impairment. Timely diagnosis is important for early intervention; currently, no tools are available to help with early diagnosis. Molecular biomarkers of cognitive impairment have been extensively studied, but clinical correlation is crucial in screening for cognitive impairment in SSD and ASD. There has been growing interest in examining the retina to scan for neurological disorders since the retina is the only part of the central nervous system that can be directly imaged non-invasively and in a timely manner. This review discusses biomarkers of cognitive impairment and their correlation to the retina in SSD and ASD. It also discusses the possible involvement of the retina and molecular biomarkers, specifically Disintegrin and metalloproteinase domain-containing protein 10 (ADAM10) and ciliary neurotrophic factor (CNTF) in the pathophysiology of SSD and ASD. A protocol for early diagnosing cognitive impairment and its severity in SSD and ASD is also suggested. This review also mentions insights into the potential use of molecular biomarkers of cognitive impairment to enhance cognitive performance in ASD and SSD and areas where more research is needed to solve the mystery of the relationship between the retina and cognitive impairment in neurodevelopmental psychiatric disorders.

## Introduction

In the past decade, there has been an increased interest in diagnosing cognitive impairment by retinal biomarkers. Psychiatric disorders involving impaired cognition can significantly impact a patient’s daily life, which increases dependency and the burden on caregivers. The psychiatric disorders involving impairment in cognition and behavior include Schizophrenia spectrum disorder (SSD), autism spectrum disorder (ASD), Parkinson’s disease, and Alzheimer’s disease (1). Cognition involves all mental processes, including knowledge, thinking, understanding, and learning. These learning mechanisms require conscious and unconscious processes through the special senses that perceive, recognize, and reason. Learning is an essential process of cognition that leads to changes in the ‘wiring’ of the brain, known as plasticity. Neural plasticity mainly occurs in childhood and involves many molecular mechanisms, including neurotransmitters, synaptic remodeling, Ca^2+^ influx, and gene expression (2, 3). Thus, cognitive impairment requires an interdisciplinary focus to deliberate the causes and provide therapy.

The retina is considered an extension of the brain and has been linked to many neurological disorders (4). Anatomically, the macula is in the center of the retina and is supplied by two special vascular systems. Histologically, it consists of ten layers: inner and external limiting membrane, photoreceptors, ganglion cells, nerve fiber layer, inner and outer plexiform layer, inner and outer nuclear layer, and pigment epithelium (5). Because brain imaging in neurological disorders shows certain abnormalities associated with specific retinal measurements, scanning the retina could be a potential non-invasive and cost-effective biomarker for early diagnosis of neuropsychiatric disorders. For example, the inner and outer retina volumes were related to the hippocampus. In contrast, the inner retina was associated with white matter, and the outer retina was associated with the grey matter of the brain (6). This indicates that the retina reflects explicitly parts of the brain and can be a target for early diagnosis of neurodevelopmental disorders. As it is known that the brain undergoes plasticity during the learning process, the retina also undergoes plasticity, which has been linked to cognitive and behavioral impairment, especially those occurring during the neurodevelopmental stages such as Schizophrenia and Autism spectrum disorders (7).

Schizophrenia spectrum disorder (SSD) and autism spectrum disorder (ASD) are neurodevelopmental disorders associated with social and communication impairment and cognitive dysfunction. While ASD is characterized by repetitive restrictive behavior, SSD involves hallucinations and delusions (8). Children with ASD are 3.5 times more likely to develop Schizophrenia compared to neurotypical children (9). Also, SSD share autistic characteristics that are sometimes confused and misdiagnosed as ASD. Historically, ASD was considered an early stage of Schizophrenia until the 1970s, when Schizophrenia and autism were finally considered as separate disorders (10). Many researchers suggest that neurobiological similarities between both disorders are reflected in brain imaging and molecular biomarkers. These changes are believed to be related to prenatal exposure to proinflammatory cytokines, contributing to the phenotypical changes observed in ASD and SSD (11). The common pathology between ASD and SSD may be reflected in the similarities observed in cognitive impairment, which include impaired visuospatial perception, visual attention and organization, memory, and language (12).

Early diagnosis is essential for early intervention to improve children’s cognitive progress and prevent further deterioration in cognitive function in SSD and ASD (13). Also, early detection of cognitive impairment would improve the patients’ and their caregivers’ quality of life and would be of high financial benefit. In this context, there is a high urge to find a non-invasive and reliable tool for early diagnosis of cognitive impairment. The retina has been tested as a site for diagnosing cognitive impairment, especially in Alzheimer’s disease patients (14). This review focuses on the molecular basis of cognition as a potential diagnostic and therapeutic tool in SSD and ASD.

## The eye and cognitive function

The relationship between retinal biomarkers and the diagnosis of cognitive function is relevant in neurodegenerative diseases, particularly Alzheimer’s disease (14). Recent clinical and molecular studies found that retinal biomarkers can be a diagnostic tool for childhood psychosis. Retinal biomarkers are non-invasive, time and cost-effective.

One of the tests used in cognitive testing is the “Reading the Mind in the Eyes” Test, which was one of the first attempts to relate the eye to cognitive impairment. This test involves a series of photographs showing different facial expressions to describe what the person in the picture is feeling depending on visual perception. This determines a person’s cognitive function based on reasoning, memory, and perception (15). Another test relating the eye to cognitive impairment is the eye-tracking test, which tests executive cognitive functions such as working memory and attention. It involves non-invasive measures of a person’s gaze and eye movement as a quantitative measurement of saccadic eye movement (16).

Recently, retinal scanning tools detected anatomical change in the retinal microvasculature, inner retinal layers, nerve fiber layer, and macular thickness detected by optical coherence tomography (OCT) and fundus photography (17, 18). The OCT is non-invasive and can objectively quantify retinal changes and potentially measure cognitive impairment.

Apart from its role in diagnosing cognitive impairment, the eye was also part of cognition intervention. For example, cognitive remediation therapy is a therapeutic tool to improve cognitive functions through plasticity, particularly in the prefrontal lobe. It proved its therapeutic outcomes, especially in bipolar and SSD. A study found that the effectiveness of cognitive remediation therapy was significant post-treatment, only in vision-related domains. Another cognitive training that depends on vision is the vision-based speed of processing, which focuses on attention and visual processing speed (19).

All this evidence supports the correlation between the eye and cognitive impairment as a diagnostic and therapeutic tool in neuropsychiatric disorders involving cognitive impairment.

## Retinal plasticity and cognitive function

The term ‘plasticity’ means the ability to be molded. Plasticity occurs in the central nervous system by potentiation (strengthening of neural connection) or depression (weakening of neural connection). Potentiation can be structural, which involves changing the neural structure as a result of learning, or functional, which occurs when a functional transfer from the damaged area of the brain to the undamaged area. In the neurodevelopmental stage, plasticity is different from those that occur in adulthood (3). The plasticity processes are faster and easier than in adulthood, where many neuron connections have undergone depression. For this reason, finding biomarkers for early detection of cognitive impairment is crucial in childhood to reach the best therapeutic outcomes and prevent cognitive decline.

Molecular plasticity involves mainly N-methyl-D-aspartate receptors (NMDARs), Ca2+/calmodulin-dependent protein kinase II (CaMKII), the cAMP-dependent protein kinase A (PKA), and the mitogen-activated protein kinase (MAPK). However, environmental factors and epigenetics play an important role in manipulating neuron plasticity (3).

Plasticity of the visual system, including the retina as part of the central nervous system, mainly occurs in the neurodevelopmental stages (7). The primary visual cortex receives sensory inputs from the left and the right eyes. A disease that affects the eyes earlier in life affects the visual cortex and the whole visual track, leading to topographical changes in the afferents of the cortex and thalamus. Children’s retina maturation occurs mainly when the eyes are open and receiving sensory stimuli (7). Sensory impairment involving vision in childhood would affect the learning process, limit potentiation, and lead to a decline in cognition (20). Animal models of light deprivation showed less plasticity in the visual cortex, showing autistic traits (21). An interesting study showed that increasing exposure to sensory, motor, and social interaction increased visual plasticity (22). This indicates that the development of the entire retina is also related to environmental settings.

## Cognitive and retina biomarkers

Many pharmacological treatments rely on the underlying molecular mechanism of a disorder. However, finding a general biomarker for cognitive impairment is difficult because many biomarkers and mechanisms are involved in different types of cognitive impairment. For example, the pathophysiology of SSD, bipolar disorder, and learning disabilities are linked to genetic factors; however, environmental factors also play a crucial role in exacerbating the symptoms that might be modified by specific therapies, including pharmacological therapy such as medications and nutrient supplements that might improve cognitive performance or delay the onset of cognitive impairment (23). Thus, finding biomarkers that are specific for cognitive impairment in SSD and ASD might aid in the discovery of pharmacological therapy for cognitive impairment. The most studied biomarkers associated with cognition are Amyloid beta peptide (Amyloid β) and tau.

Amyloid bet is formed by the proteolytic process by secretases (beta and gamma) of its precursor protein (APP), a principal protein with neurodevelopmental and neuro-signaling functions. The accumulation of extracellular Amyloid Beta misfolding causes intracellular deposition of tau protein, leading to cognitive impairment. Tau protein is essential for microtubule assembly and stability and is regulated by phosphorylation. Hyperphosphorylation of tau protein causes neuron apoptosis, leading to memory loss. The deposition of Amyloid Beta proteins causes neurotoxicity that leads to the formation of plaques in the brain, which is found in dementia and is the hallmark of Alzheimer’s disease (24). The binding of Amyloid Beta to various receptors, such as glutamate, lipid, and N-methyl-D-aspartic acid (NMDAR), leads to neurotoxicity, which induces oxidative stress and mitochondrial dysfunction. The pathophysiology of Amyloid Beta formation and binding to various receptors in dementia is poorly understood, and no drugs have been discovered yet that can reverse this process. However, some of these receptors have been a pharmacological target to prevent or reduce the acceleration of cognitive impairment in children and delay the onset and speed of memory loss in adults (25). Recent therapeutic approaches are more related to preventing the formation of Amyloid Beta and tau protein.

Interestingly, recent studies found that cognitive dysfunction and retinal degeneration share biomarkers. These biomarkers include Disintegrin and metalloproteinase domain-containing protein 10 (ADAM10) and ciliary neurotrophic factor (CNTF). The ADAM10 is part of the ADAM protein family with a catalytic function encoding the alpha-secretase gene. When APP is cleaved by alpha-secretase, it prevents Amyloid Beta production and reduces memory loss risk. The possible molecular mechanism for plaque formation in cognitive disorders such as Alzheimer’s is that reduced ADAM10 leads to decreased alpha-secretase. This will allow beta and gamma-secretase to cleave APP, increasing Amyloid Beta and plaque formation in the brain (26).

**Figure 1:**
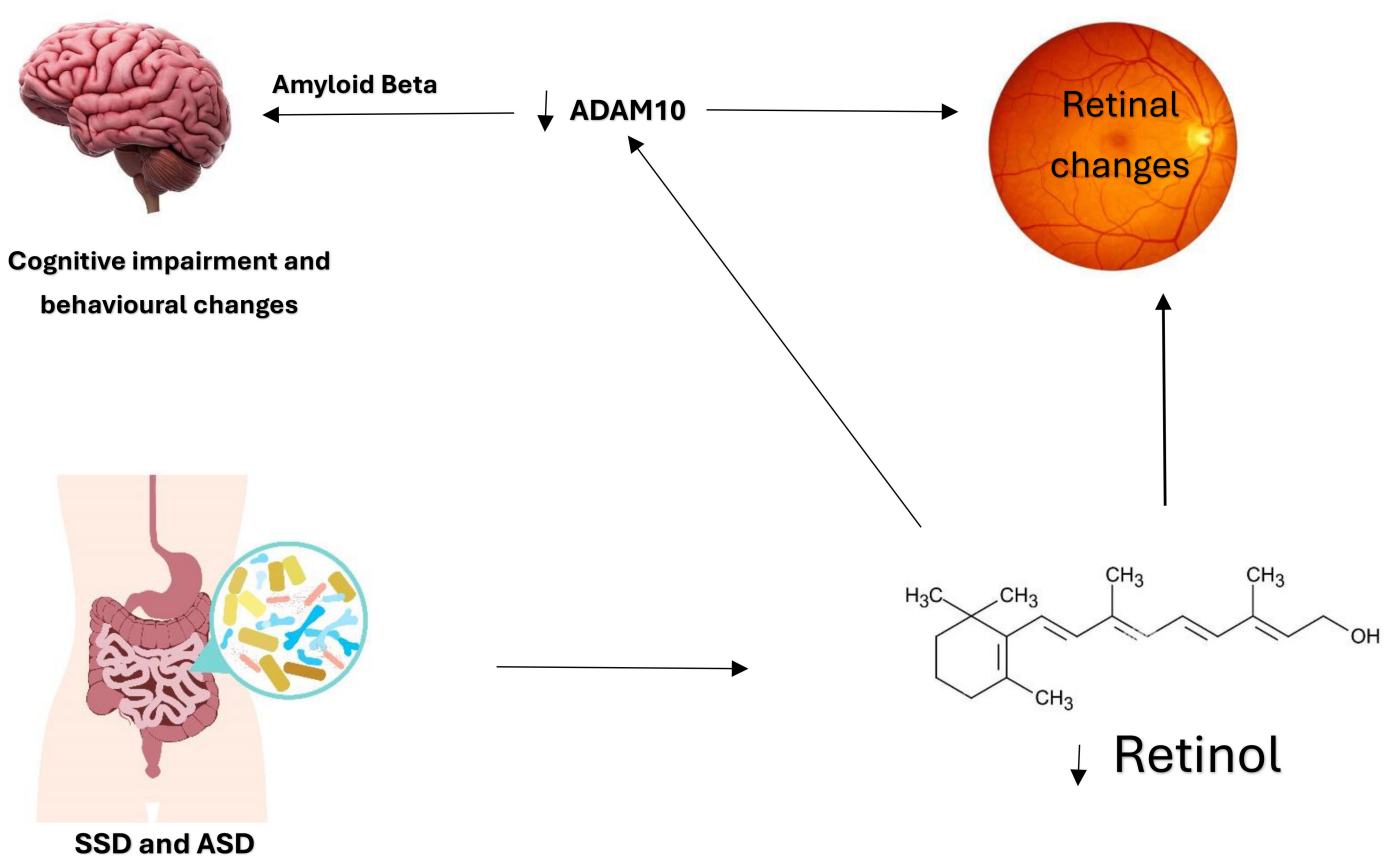
The Gut-Brain Axis and the possible involvement of the eye. Dysfunctional gut and microbiota in ASD and SSD lead to reduced vitamin A absorption and activation. The active form of vitamin A is retinoic acid, essential for retinal development and ADAM10 activation. The relationship between ADAM10, the retina, and retinoic acid is noteworthy. Reduced retinoic acid affects retinal development, and ADAM10 levels lead to Amyloid Beta formation and cognitive impairment. This relationship is further demonstrated in **Figure 2**. The development of the retina requires vitamin A and ADAM10. Hence, it is speculated that vitamin A deficiency directly affects retinal development through retinoic acid receptors and indirectly by downregulating ADAM10.

Levels of ADAM10 are reduced in mild cognitive impairment, indicating its role as an early indicator of cognitive impairment (27, 28). In addition to its role as a cognitive impairment biomarker, ADAM10 is crucial for normal retina development and was found to be decreased in retinal degenerative disorders. The possible connection between ADAM10 and the retina is Vitamin A. ADAM10 can be naturally induced by retinoic acid, which is the active product of retinol (Vitamin A) (26). The absorption and activation of vitamin A require healthy gut and gut microbiota, and then the final activation and storage of vitamin A occur in the liver. Vitamin A deficiency was linked to cognitive impairment. Retinoic acid reduces Amyloid Beta formation in Alzheimer’s, suggesting its possible therapeutic role in cognitive impairment (29). This relationship may be involved in what is known as the gut-brain axis. An impaired gut function might affect the availability of vitamin A and reduce retinoic acid, leading to the downregulation of ADAM10, which produces Amyloid Beta, leading to retinal degeneration and cognitive impairment (**Figure 1**). Treating patients with cognitive impairment with vitamin A would upregulate ADAM10 and prevent Amyloid Beta formation.

A study reported that Alzheimer’s can be identified through the retina as an early indicator of the disease (30). Patients with deposition of Amyloid Beta induced by oxidative stress in the retina had decreased levels of ADAM10, suggesting the direct effect of ADAM10 on the Amyloid Beta production process (31, 32). Amyloidosis was found in the retina of Alzheimer’s disease patients, affecting their vision, and was positively related to cognitive decline (33). Treating with an exogenous ADAM10 prevents and might even reverse the amyloidogenic pathways, indicating a possible therapeutic role of ADAM10 for cognitive disorders (34).

The CNTF is a neurotrophic factor with neuroprotective effects usually found in the early stages of neural injury. It is a member of the interleukin 6 (IL-6) family, a member of neuropoietic cytokines. CNTF is one of the most studied neurotrophic factors in the eye, and it has many neuroprotective effects on photoreceptors, retinal nerve fiber, and ganglion (35). Exogenous CNTF reversed and prevented neural degeneration in many neurodegenerative diseases (36). Injecting the retina with CNTF reverses retinal degeneration and promotes photoreceptor plasticity (37). Injecting animal models of neurodegenerative diseases with CNTF reversed behavioral symptoms of the disease and provided neural protection and sparing as a potential therapy for the disease.

In addition, long-term delivery of encapsulated CNTF reversed cognitive impairment in animal models of Alzheimer’s disease (38). Similar technology was used in patients with retinal degeneration in a randomized clinical trial, which showed its effectiveness in slowing the progress of retinal degeneration (39). We speculate that long-term delivery of encapsulated CNTF would improve cognitive performance in neurodevelopmental psychiatric disorders and slow cognitive decline in neurodegenerative psychiatric disorders. Introducing CNTF in the early stages of cognitive impairment improves results by preventing Amyloid Beta and tau proteins from accumulating. It also promotes synaptic and neural function, leading to neural protection and plasticity. The CNTF administration is challenging since it does not cross the blood-brain barrier and has a low bioavailability. In addition, CNTF rescues cognitive impairment in a dose-dependent manner, so bioavailability is essential for achieving cognitive repair. Peptide 021 (P021) mimics CNTF and improves cognitive function and retinal plasticity by increasing brain-derived neurotrophic factor (BDNF) expression. It can also be taken orally (40). Unlike the ADAM10, which is best taken in the early stages of cognitive impairment, the CNTF can rescue severe and late stages of cognitive impairment in Alzheimer’s disease. Although studies reported that CNTF rescues cognitive impairment by increasing the expression of BDNF, many other studies showed that injecting BDNF was ineffective in cognitive impairment, suggesting that CNTF has additional pathways to rescue cognitive impairment in addition to increasing BDNF expression (40).

Given the strong association between retinal dysfunction and cognitive impairment, we will further discuss this association in schizophrenia and autism spectrum disorders.

## Cognitive impairment and the retina in schizophrenia

The retina is the only part of the central nervous system that can be directly imaged. Schizophrenia patients show many retinal changes, which makes retina imaging one of the most recent, reliable, and non-invasive biomarkers for early diagnosis and prognosis in SSD. In a large study that examined the retina of 485 patients with Schizophrenia, they found that SSD patients had thinner ganglion cell-inner plexiform layers compared to neurotypical controls (41). These retinal changes were structural, measured by OCT, and functional, measured by electroretinography (ERG), which were found to be related to the severity of cognitive impairment in SSD (42).

Specifically, a thinner retinal nerve fiber layer was associated with the severity of cognitive impairment in SSD, but central macular thickness was correlated to acute SSD (43–45). Changes detected by OCT in SSD were unrelated to other co-morbidities, which strongly indicates the correlation between SSD and these retinal changes (46). The chronicity of SSD also shows specific retinal morphology, which can indicate the severity of the disease (47, 48). The changes in the retina in SSD patients were also significant regardless of ethnicity and family history of retinal disorders (46, 48). Another finding is that the macular thickness and volume were decreased in patients with SSD compared with age-matched controls, indicating accelerated aging in the retina of SSD patients, and the severity of these retinal changes was associated with the severity of cognitive impairment (49).

Implementing a specific eye screening protocol for SSD is essential for early diagnosis and might also determine the progression of cognitive impairment in SSD. Despite this mounting evidence of the correlation between the retina and cognitive impairment in SSD, studies on the pathways leading to these findings are scarce. Research is needed to test this relationship at the molecular level to develop a biomarker for early diagnosis, prognosis, and pharmacological therapy for cognitive impairment in SSD.

Molecular biomarkers of cognitive impairment and retinal dysfunction were significantly reported in Schizophrenia. CNTF is decreased in Schizophrenia, and it was significantly decreased as the severity of cognitive impairment increased. Also, reduced CNTF levels were associated with a reduction in retinal nerve fiber layers in schizophrenic patients (50). The retinal thickness is detected in the early stages of the disease, which, when detected early, can prevent further changes in the retina. The decreased level of CNTF might contribute to a therapeutic intervention to prevent or reverse retinal dysfunction that might improve or prevent cognitive impairment in SSD. Another retinal biomarker is the vascular endothelial growth factor (VEGF), a known biomarker for retinal ischemia correlated with neuropsychiatric disorders, including SSD. VEGF was reported to be elevated in SSD patients with thinner retinal nerve fiber layers and was also positively correlated with cognitive impairment and the chronicity of the disease (44).

In addition, ADAM10 was found to be downregulated in SSD (51). Because ADAM10 is essential for the physiological functions of neurons, the decreased ADAM10 in SSD might contribute to the excitatory/inhibitory imbalance and plasticity, which are the main regulators of behavior and cognition. The expression of ADAM10 is affected by retinol. This might explain why embryogenic retinol deficiency affects the brain’s normal development and leads to synaptic dysregulation and plasticity, which might be a risk factor for SSD. Thus, pharmacotherapy or a diet containing retinol might enhance cognitive performance in SSD (52). Patients with SSD are known to have a dysfunctional gut, which might have affected the gut-brain axis, possibly by vitamin A deficiency (**Figure 1**). Decrease absorption of vitamin A due to gut or gut microbiota dysfunction, which is known in SSD and ASD, might lead to reduced retinoic acid reaching the retina and decreasing levels of ADAM10, which was also found to be reduced in ASD and SSD, with associated cognitive impairment. Hypovitaminosis in SSD can be caused by the antipsychotic drugs that affect the metabolism of certain vitamins such as A, B, and D, which might affect neural function and can be associated with poor cognitive performance in psychiatric patients (53).

## Cognitive impairment and the retina in autism

Many biomarkers in autism were related to the severity of autistic behavior and cognitive impairment, which involves neural pathways correlated to the pathophysiology of ASD (54–56). As studies reported that blindness occurring in late childhood might lead to Schizophrenia, evidence from children and blind animal models reported that blindness in early life or at birth, before visual cortex plasticity and maturation, leads to autistic traits. Retina in ASD and its relation to the severity of autism and cognitive function has not been thoroughly studied. Recent evidence reported specific morphology in the retina of ASD patients as a possible biomarker of the disorder. Recently, software has been developed to detect autism using artificial intelligence by analyzing retinal images (57). Molecular studies in ASD correlated retinal dysfunction to autistic traits, and some pharmacotherapy on animal models of autism showed reduced autistic behavior and improved cognitive performance (58).

**Figure 2:**
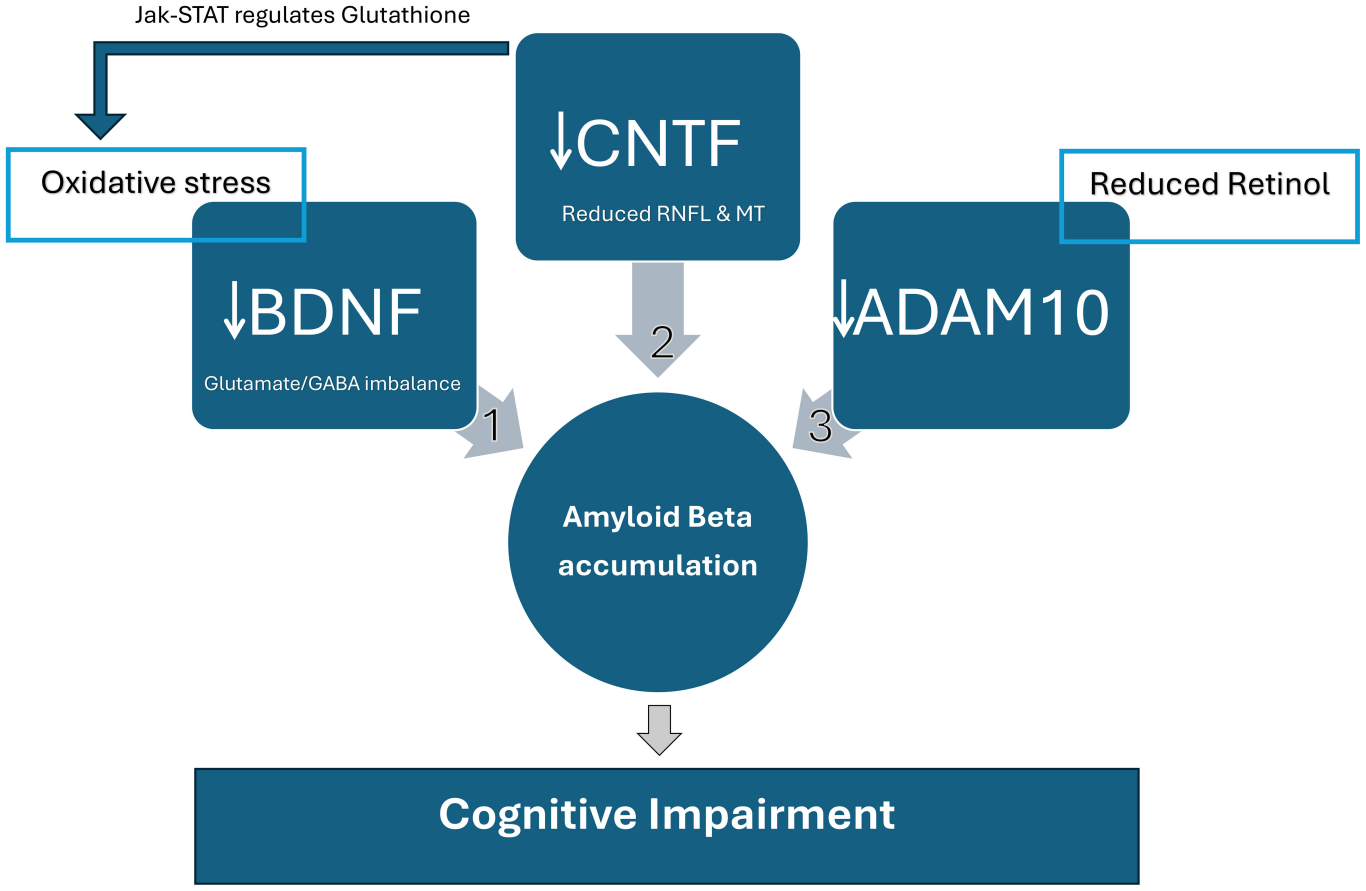
Relationship between molecular biomarkers affecting cognitive function in SSD and ASD. These biomarkers can be an early diagnosis marker and severity and prognosis indicator. 1) BDNF: Factors such as oxidative stress lead to the depletion of Brain-derived neurotrophic factor (BDNF) and accumulation of Amyloid Beta in ASD and SSD. Reduced BDNF leads to behavioral changes and cognitive impairment caused mainly by Glutamate/GABA imbalance. 2) CNTF: Reduced retinal nerve fiber layers (RNFL) and macular thickness (MT) are associated with low Ciliary neurotrophic factor (CNTF) levels and the severity of cognitive impairment in SSD. It is suggested that SSD and ASD have led to the abnormalities in RNFL and MT as part of the pathophysiology of both disorders. There are two pathways in which CNTF affects cognitive function. One is through forming Amyloid Beta, and the other is through the Janus Kinase (JAK)-Signal Transducers and Activators of Transcription (STAT) pathway. CNTF is Glutathione’s main regulator, which metabolically restores retinal degeneration and reduces oxidative stress, improving BDNF. The Jak-STAT stimulation by CNTF directly leads to retinal re-generation and restores neural function, enhancing cognition. 3) ADAM10: ADAM10 is reduced in Vitamin A deficiency, as demonstrated in **Figure 1**. It was also associated with retinal degeneration, which affects cognitive functions. Reduced ADAM10 due to vitamin A deficiency or retinal degeneration leads to APP cleavage by beta and gamma-secretase, generating Amyloid Beta accumulation. ADAM10 cleaves APP by alpha-secretase, preventing Amyloid Beta formation and rescuing cognitive function.

Biomarkers such as CNTF, Amyloid Beta, tau protein, ADAM10, BDNF, and oxidative stress are dysregulated in ASD (59). These biomarkers shown in (**Figure 2**) are possibly connected in a way that we found to be worth investigating. Since ADAM10 is essential for neurons, synapses, brain, and retinal development, impaired ADAM10 in the neurodevelopmental stage might lead to neurodevelopmental disorders characterized as cognitive impairment. Oxidative stress was also reported in ASD, and it is one of the factors that impair ADAM10 function, which might contribute to the pathophysiology of cognitive impairment in ASD patients. Vitamin A might also have a role in ADAM10 dysregulation in ASD. Studies reported that vitamin A supplementation improved ASD symptoms (60). We speculate that improved ASD symptoms might be through the ADAM10 pathway, which physiologically improves excitation/inhibition balance and cognitive performance (**Figure 1**).

## Biomarkers as a possible diagnostic and therapeutic tool

Neurodevelopmental disorders need careful attention because delayed or missed diagnosis leads to lifelong complications that affect not only the child but also the caregivers, siblings, and the community and add financial burden to the healthcare system. Studies have shown that the best intervention for cognitive impairment in children is early diagnosis and intervention. To achieve early diagnosis, we suggest that the screening tool meet specific criteria: it should be non-invasive, available, affordable, and time-efficient. The retina can be scanned by OCT, which is non-invasive, readily available, affordable, and time-efficient. The retina shows specific morphology in SSD and ASD, making it a good candidate diagnostic and severity indicator biomarker for cognitive impairment in ASD and SSD. The shared biomarkers between retinal and cognitive dysfunction are ADAM10 and CNTF, as they show promising diagnostic and therapeutic targets for cognitive impairment in neurodevelopmental psychiatric disorders interventions for multiple reasons: 1- Early diagnosis, as decreased CNTF and ADAM10 were detected in early stages of cognitive impairment in Alzheimer’s Disease patients and acute SSD. 2- Predict possible involvement of retina anatomical changes and cognitive impairment. 3- Fulfill the suggested diagnostic tool criteria as it is affordable, reliable, time efficient, and available but minimally invasive as it requires blood samples from the patients. 4- The analogs of CNTF and ADAM10 are available for clinical use as vitamin A supplements can upregulate ADAM10 and have been extensively studied in SSD and ASD. 5- Animal studies have reported the effectiveness of CNTF and ADAM10 in cognitive impairment.

## Future insight

Molecular psychiatry is a promising field in neurodevelopmental psychiatric disorders, offering a unique opportunity to diagnose neuropsychiatric disorders in an early preclinical stage before patients show behavioral and cognitive symptoms. The most relevant molecular biomarkers are those related to retinal structural changes. We strongly suggest studying the relationship between the retina OCT and correlating it to clinical cognitive tests and molecular biomarkers to establish early diagnostic criteria for SSD and ASD as a specific and comprehensive tool at the preclinical stage. We also suggest studying this relationship using animal models of SSD and ASD to find the molecular pathway affecting the retina-brain axis leading to cognitive impairment, specifically in SSD and ASD.

## Conclusion

Early diagnosis and treatment are essential to achieve the best clinical outcomes in Schizophrenia and Autism spectrum disorders. This can be achieved by finding the most appropriate molecular biomarker that can be the target for pharmacological intervention. Recently, a significant correlation was found between specific retinal structural changes and the severity of cognitive impairment in SSD and ASD. Hence, finding molecular biomarkers is essential to explain the relationship and molecular pathways between the retina and cognitive impairment. The suggested biomarkers are CNTF and ADAM10, which have been clinically investigated in many retinal and cognitive disorders, creating a new pharmacological research venue for enhancing cognitive performance in SSD and ASD.

## Data Availability

The original contributions presented in the study are included in the article/supplementary material. Further inquiries can be directed to the corresponding author.
